# *Wolbachia* supergroup A in *Enoplognatha latimana* (Araneae: Theridiidae) in Poland as an example of possible horizontal transfer of bacteria

**DOI:** 10.1038/s41598-024-57701-y

**Published:** 2024-03-29

**Authors:** Edyta Konecka, Paweł Szymkowiak

**Affiliations:** 1 Department of Microbiology, Faculty of Biology, Adam Mickiewicz University, Poznań, Uniwersytetu Poznańskiego 6, 61-614 Poznań, Poland; 2 Department of Animal Taxonomy and Ecology, Faculty of Biology, Adam Mickiewicz University, Poznań, Uniwersytetu Poznańskiego 6, 61-614 Poznań, Poland

**Keywords:** Bacterial genes, Phylogenetics

## Abstract

*Wolbachia* (phylum Pseudomonadota, class Alfaproteobacteria, order Rickettsiales, family Ehrlichiaceae) is a maternally inherited bacterial symbiont infecting more than half of arthropod species worldwide and constituting an important force in the evolution, biology, and ecology of invertebrate hosts. Our study contributes to the limited knowledge regarding the presence of intracellular symbiotic bacteria in spiders. Specifically, we investigated the occurrence of *Wolbachia* infection in the spider species *Enoplognatha latimana* Hippa and Oksala, 1982 (Araneae: Theridiidae) using a sample collected in north-western Poland. To the best of our knowledge, this is the first report of *Wolbachia* infection in *E. latimana*. A phylogeny based on the sequence analysis of multiple genes, including 16S rRNA, *coxA*, *fbpA*, *ftsZ*, *gatB*, *gltA*, *groEL*, *hcpA*, and *wsp* revealed that *Wolbachia* from the spider represented supergroup A and was related to bacterial endosymbionts discovered in other spider hosts, as well as insects of the orders Diptera and Hymenoptera. A sequence unique for *Wolbachia* supergroup A was detected for the *ftsZ* gene. The sequences of *Wolbachia* housekeeping genes have been deposited in publicly available databases and are an important source of molecular data for comparative studies. The etiology of *Wolbachia* infection in *E. latimana* is discussed.

## Introduction

*Wolbachia*—a maternally inherited bacterial symbiont—is widespread^1–5^ and exhibits a spectrum of interactions with its hosts, ranging from mutualistic to parasitic^6^ and has the ability to manipulate host reproduction to enhance its own transmission^7^. Studies have shown that *Wolbachia* can exert both immediate and long-term effects on their hosts. Not only it can act without affecting the host genome^8^, but, importantly, it can also induce changes in the genome of the invertebrate^9^, which may be passed on to the next generation with all the implications of these changes^10^. Therefore, by studying the occurrence of endosymbionts, one can gain a comprehensive understanding of its symbiotic relationships with various hosts. A holistic view of the eukaryotic organism as a holobiont not only has a cognitive aspect, but also allows to trace the path of transmission of microbes and determine their roles within the hosts. This perspective is particularly relevant when studying the interactions between *Wolbachia* and its host organisms, as these interactions can be highly intricate and dynamic. Firstly, the same bacterial strain may exert various effects depending on the arthropod genotype^11^, and secondly, the response of an invertebrate host to infection with different *Wolbachia* strains can also vary^12^. Additionally, the *Wolbachia*’s associations with other bacteria within the host microbiota play a significant role in shaping the activity and functions of these microbial communities. By modifying the composition of the microbial community, *Wolbachia* can indirectly affect important aspects of the host’s physiology, such as nutrition or pathogen resistance^13^.

*Wolbachia* constitutes an important force in the evolution^14^, biology^15^, and ecology^16^ of invertebrate hosts. It can cause sex-ratio distortion by inducing several phenotypes in hosts such as feminization^17,18^, parthenogenesis^19^, male-killing or cytoplasmic incompatibility^20^. The microorganism also enhances insect reproduction by providing biotin and vitamin B^21,22^, leading to increased egg production^23^ and improved fecundity of invertebrates^24^. The bacterium may also exert other effects on the host in addition to those related to reproduction. For example, *Wolbachia* can prevent infections caused by fungal^8^ or bacterial pathogens^25^, and reduce pathogenic viral loads in various arthropod species^26,27^. It can also decrease host susceptibility to different chemical pesticides^28,29^, which may be associated with changes in metabolism, detoxification gene expression or immune responses in bacterial hosts^30^.

On the basis of the phylogeny of housekeeping genes^31–34^ or whole-genome typing methods^35,36^, the genus *Wolbachia* has been divided into supergroups and labelled with letters of the alphabet^37^. An examplary set of genes comprises: *coxA* coding for cytochrome c oxidase, *gatB* coding for glutamyl-tRNA(Gln) amidotransferase, *hcpA* coding for conserved hypothetical protein, *ftsZ* coding for prokaryotic cell division protein, *fbpA* coding for fructose-bisphosphate aldolase, and additionally the *wsp* gene encoding *Wolbachia* surface protein^31^, *groEL* encoding 60 kDa heat-shock protein, and *gltA* coding for citrate synthase^32–34^. Sequence-based analysis of bacterial housekeeping genes as a set of genotyping markers can identify and discriminate closely related strains and accurately determine genetic divergence between them^38^.

Insects are the most comprehensively studied group of invertebrates in terms of the occurrence of endosymbiotic bacteria^39^. However, unlike insects, there are groups of invertebrates about which knowledge about the frequency and diversity of intracellular microbes is significantly more limited. Spiders are an example. Similarly, the etiology of infection, host specificity and effects of endosymbiotic bacteria in spiders are poorly characterized. Nevertheless, some literature data suggest that spiders may have more diverse microbiome than insects^40,41^, indicating the potential presence of novel, undiscovered taxa of microorganisms^41–44^. Spiders are one of the most successful terrestrial colonizers, but the data regarding their endosymbiotic relationships are scarce. Therefore, searching for, describing and understanding the presence of these organisms in spiders is required, especially since it would be interesting to elucidate whether their microbiomes have contributed to the evolutionary success of spiders.

The presence of *Wolbachia* in spiders has been observed relatively rarely^45–58^; however, most studies have examined only a few individuals of spiders of certain species^40,41,59–63^. Bacterial strains occurring in spiders have been classified in supergroup A and B^44^ together with *Wolbachia* infected insects, isopods, and mites—carriers of bacteria from supergroup B^37^. The extent of phenotypic effects induced by microbial endosymbionts in spiders remains largely unknown. Exceptions to this limited knowledge include cases where *Wolbachia* has been associated with sex ratio imbalances in certain spider species. In *Oedothorax gibbosus* (Blackwall, 1841), the killing of male embryos is most likely a manipulative effect of *Wolbachia*^50^; in *Mermessus fradeorum* (Berland, 1932), *Wolbachia* is suspected of causing cytoplasmic incompatibility and feminization^64^; and lastly, in *Pityohyphantes phrygianus* (C. L. Koch, 1836), *Wolbachia* may influence female post-copulatory behavior and sex ratio^48^. Another effect caused by *Wolbachia*, not directly related to reproduction, was observed in the spider *Hylyphantes graminicola* (Sundevall, 1830), where the bacteria beneficially affected host metabolism^30^, leading to increased enzyme activity and nutrient availability, which contributed to a higher survival rate of the spider under stress^65^.

The available data on the spread of *Wolbachia* in spiders are still insufficient, and the diversity of bacterial strains determined by Multilocus Sequence Typing (MLST) in this group of arthropods from Poland is unknown. Therefore, we decided to pursue the issue of intracellular bacteria in spiders. The aim of our study was to determine the distribution and molecular characterization of *Wolbachia* in these invertebrates, which may contribute to better understanding of host-endosymbiont associations. Here, we report the first detection of *Wolbachia* in the spider *Enoplognatha latimana* Hippa and Oksala, 1982 (Araneae: Theridiidae). The *Wolbachia* strain identified in this spider was examined using MLST and *wsp* gene analyses. Furthermore, we discuss the etiology of *Wolbachia* infection in *E. latimana*.

## Materials and methods

### Sampling of spiders

Thirty-four *E*. *latimana* adult specimens, three juvenile forms, and two egg sacs were collected from nine different locations in the Wielkopolska Voivodeship: (1) coordinates: N 52.46136, E 16.94071; collection date: September 2021; (2) coordinates: N 52.49315, E 16.88068; collection date: July 2021; (3) coordinates: N 52.49315, E 16.88068; collection date: July 2021; (4) coordinates: N 52.49273, E 16.87891; collection date: July 2021; (5) coordinates: N 52.34225, E 18.47713; collection date: August 2021; (6) coordinates: N 52.18284, E 17.746977; collection date: July 2021; (7) coordinates: N 52.64025, E 19.12977; collection date: June 2021; (8) coordinates: N 52.63449, E 19.32619; collection date: June 2021; and (9) coordinates: N 52.47559, E 16.92671; collection date: July 2021.

Three adult male spiders, one adult female spider, and one juvenile form were collected from the same locality characterized in Table 1. The spiders were collected using a sweep net and immediately placed in 96% ethanol. Each specimen was examined for the presence of *Wolbachia*, as described below. The spiders were also examined for the presence of parasitoid insects by microscopic observation.

**Table 1:** Identified *Wolbachia* gene sequences in the host *Enoplognatha latimana* and sampling site localities.

Sample collection locality	*Wolbachia* gene sequence (GenBank accession number, length)
Area with shrubs, shaded with a rich undergrowth of herbaceous vegetation, near municipal waste landfill of the city of Poznań, Wielkopolska Voivodeship	16S rRNA (OR220066, 1335 bp) *coxA* (OR227583, 410 bp) *fbpA* (OR227584, 417 bp) *ftsZ* (OR227585, 462 bp) *gatB* (OR227586, 398 bp) *gltA* (OR227587, 552 bp) *groEL* (OR227588, 441 bp) *hcpA* (OR227589, 416 bp) *wsp* (OR227590, 536 bp)
Coordinate	
N 52.4931526184082, E 16.88068199157715	
Collection date	
01.07.2021	

### *Wolbachia* detection

Total DNA was isolated from individual specimens using silica membranes from the commercial Genomic Mini kit for universal genomic DNA isolation (A&A Biotechnology, Gdansk, Poland) according to the manufacturer’s instruction. *Wolbachia* was identified by PCR using the following *Wolbachia*-specific primers: 553F_W (5′-CTTCATRYACTCGAGTTGCWGAGT-3′) and 1334R_W (5′-GAKTTAAAYCGYGCAGGBGTT-3′)^66^, which amplified a 781-bp product of the 16S rRNA gene. The PCR amplification was as follow: 94 °C for 2 min, 35 cycles at 94 °C for 30 s, 62 °C for 30 s, and 72 °C for 45 s, and final elongation at 72 °C for 10 min^66^.

### Analysis of *Wolbachia* genes

Molecular characterization of *Wolbachia* was based on sequence analysis of housekeeping genes: 16S rRNA, *coxA*, *fbpA*, *ftsZ*, *gatB*, *gltA*, *groEL*, *hcpA*, and additionally *wsp*. Two PCR reactions were conducted for the amplification of the 16S rRNA gene sequence. The first reaction utilized the specific primer EHR16SD^67^ along with the universal eubacterial primer 1513R^68^. The second reaction employed the specific primer EHR16SR^67^ along with the universal eubacterial primer 63F^69^. Other housekeeping genes included in the analysis were: *gatB* (glutamyl-tRNA(Gln) amidotransferase), *coxA* (cytochrome c oxidase), *hcpA* (conserved hypothetical protein), *ftsZ* (cell division protein), *fbpA* (fructose bisphosphate aldolase), *wsp* (*Wolbachia* surface protein)^31^, *gltA* (citrate synthase)^32^, and *groEL* (60-kDa heat-shock protein)^70^. The primer sequences and PCR amplification conditions are presented in Supplementary Table S1. PCR products were analyzed by electrophoresis on a 1.5% NOVA Mini agarose gel (Novazym) with a Nova 100 bp DNA Ladder (Novazym), sequenced using BigDye Terminator v3.1 with ABI Prism 3130XL (Applied Biosystems) and compared to the GenBank sequence data (International Nucleotide Sequence Database Collaboration) using BLASTn. *Wolbachia* gene sequences were deposited in GenBank under the accession numbers listed in Table 1.

### MLST and phylogenetic analysis using *wsp* and *ftsZ* genes

MLST analysis was performed targeting the following eight loci: 16S rRNA, *coxA*, *fbpA*, *ftsZ*, *gatB*, *gltA*, *groEL*, and *hcpA*. Individual sequences of *Wolbachia* genes were aligned with sequences of different *Wolbachia* supergroups deposited in the GenBank database. Phylogenetic trees based on MLST were constructed for single genes, as well as concatenated alignments of the eight bacterial loci, using the maximum-likelihood method in MEGA 11 software^71^. Additionally, the sequences of *Ehrlichia* sp. were included as an outgroup. The NCBI accession numbers of the sequences used in the phylogenetic analysis are presented in Supplementary Figs. S1–S8. Sequence alignments were generated using CLUSTAL W software^72^. The jModelTest 2 software^73,74^ was applied to select the appropriate sequence evolution model. The HKY + G model was selected for 16S rRNA, *coxA*, and *fbpA* sequences, while the TrN + I + G model was chosen for the *ftsZ* sequence data; the GTR + G model was used for *gatB*, *gltA*, and for the concatenated sequence data of eight genes (16S rRNA, *coxA*, *fbpA*, *ftsZ*, *gatB*, *gltA*, *groEL*, and *hcpA*); the TrN + G model was selected for sequences available for the *groEL* and *hcpA* genes. Genetic recombination between strains was detected using the φ test implemented in the SplitsTree4 software^75^. The maximum likelihood bootstrap support was determined using 1000 bootstrap replicates.

The *wsp* gene, due to its relatively fast evolutionary rate, experiences significant recombination and diversifying selection, making it unreliable for strain characterization when used alone. However, it can be used as an additional optional strain marker to complement the MLST information^31^. The *wsp* gene sequence of *Wolbachia* from *E. latimana* was aligned with corresponding sequences of *Wolbachia* supergroups A and B deposited in GenBank. Additionally, an outgroup of *Wolbachia* supergroup D sequence was included. The NCBI accession numbers for the sequences used for phylogenetic analysis are shown in Fig. 3. The phylogenetic tree of the *wsp* gene was reconstructed using the same parameters as described above. The GTR + I + G model was selected for the *wsp* sequence.

The *ftsZ* gene, which is involved in the regulation of bacterial cell division, contains highly conserved regions^76^. This characteristic makes it suitable for conducting fine-scale phylogenetic analysis within a bacterial genus^77^. A phylogenetic network was constructed based on the *ftsZ* gene sequences of *Wolbachia* using neighbor-net algorithm distance estimates in SplitsTree4. Unlike traditional phylogenetic trees, a phylogenetic network allows for visualization of multiple connections among examined sequences, which can represent recombination events^75,78^.

Additionally, the *coxA*, *fbpA*, *ftsZ*, *gatB*, and *hcpA* genes were compared with sequences in the PubMLST database (https://pubmlst.org) for generating a MLST allelic profile, determining the sequence type (ST) and the clonal complex.

## Results

All collected specimens were screened for the occurrence of *Wolbachia* and the bacterium was detected in one female only. The infected female was collected together with three adult males and one juvenile from the same population, which were tested negative for *Wolbachia* infection. In addition, no parasitoid insects were observed upon microscopic examination.

We have successfully detected *Wolbachia* in *E. latimana*, marking the first documented occurrence of this bacterium in this spider species. Our analysis involved amplification of the *wsp* gene and eight housekeeping genes (16S rRNA, *coxA*, *fbpA*, *ftsZ*, *gatB*, *gltA*, *groEL*, and *hcpA*) of *Wolbachia* (Table 1).

### Comparison of gene sequences

*Wolbachia* 16S rRNA, *coxA*, *fbpA*, *ftsZ*, *gatB*, *gltA*, *groEL*, and *hcpA* housekeeping gene sequences housekeeping gene sequences were compared with sequences deposited in GenBank from various invertebrate hosts using BLASTn. The gene sequences of *Wolbachia* from *E. latimana* showed the highest identity with *Wolbachia* from other spiders representing the order Araneae, as well as from insects from the orders Diptera and Hymenoptera.

The 16S rRNA gene sequence of bacteria from *E*. *latimana* showed the highest identity with *Wolbachia* sequence from dipteran insects *Aedes albopictus* (Skuse, 1894) and *Drosophila* *sturtevanti* Duda, 1927 deposited in GenBank under accession numbers CP101657 and CP050531, respectively. Sequence query coverage was 99% and the identity was 99.55%. We also compared the 16S rDNA sequences of *Wolbachia* infecting *E. latimana* and *Enoplognatha ovata* (Clerck, 1757) (accession no. EU333941), since both hosts represented the same genus of spiders. The identity of these sequences was 99.76% with query coverage of 62%.

Sequence analysis of the *coxA* amplicon using BLASTn showed the highest identity of 98.78% with 100% query coverage with *Wolbachia coxA* from the spider *Mesida yini* Zhu, Song and Zhang, 2003 deposited in GenBank under accession no. KX169178. The highest identity (100% with 100% query coverage) was observed between *Wolbachia fbpA* sequences from *E. latimana* and the spider *Leucauge celebesiana* (Walckenaer, 1842) (accession no. KX380749). The *ftsZ* sequence showed the highest identity of 98.48% with 99% query coverage with *Wolbachia* gene from *D.* *sturtevanti* and the hymenopteran insect *Camponotus sayi* Emery, 1893 deposited in GenBank under accession numbers CP050531 and DQ266387, respectively. The sequence of the *gatB* gene of *Wolbachia* from *E. latimana* exhibited the highest identity (100%) to the gene of *Wolbachia* from the spider *Metellina ornata* (Chikuni, 1955) (accession no. MN202032). The highest identity of 98.36% with 99% query coverage was observed between the sequences of the *gltA* amplicon detected in *Wolbachia* from *E. latimana* and the dipteran insect *Sicus ferrugineus* (Linnaeus, 1761) (accession no. OX366370). Sequence analysis of the *groEL* amplicon using BLASTn showed the highest identity of 95.05% with 100% query coverage to *Wolbachia groEL* from the spider *O*. *gibbosus* deposited in GenBank under accession no. OW370537. The highest identity (97.6% with 100% query coverage) was observed between the *Wolbachia hcpA* sequences from *E. latimana* and the hymenopteran insect *Camponotus pennsylvanicus* (De Geer, 1773) (accession no. CP095495). The *wsp* sequence showed the highest identity of 99.25% with 99% query coverage with the gene of *Wolbachia* from the spider *Trichonephila clavata* (L. Koch, 1878) deposited in GenBank under accession no. EF612772.

**Figure 1:**
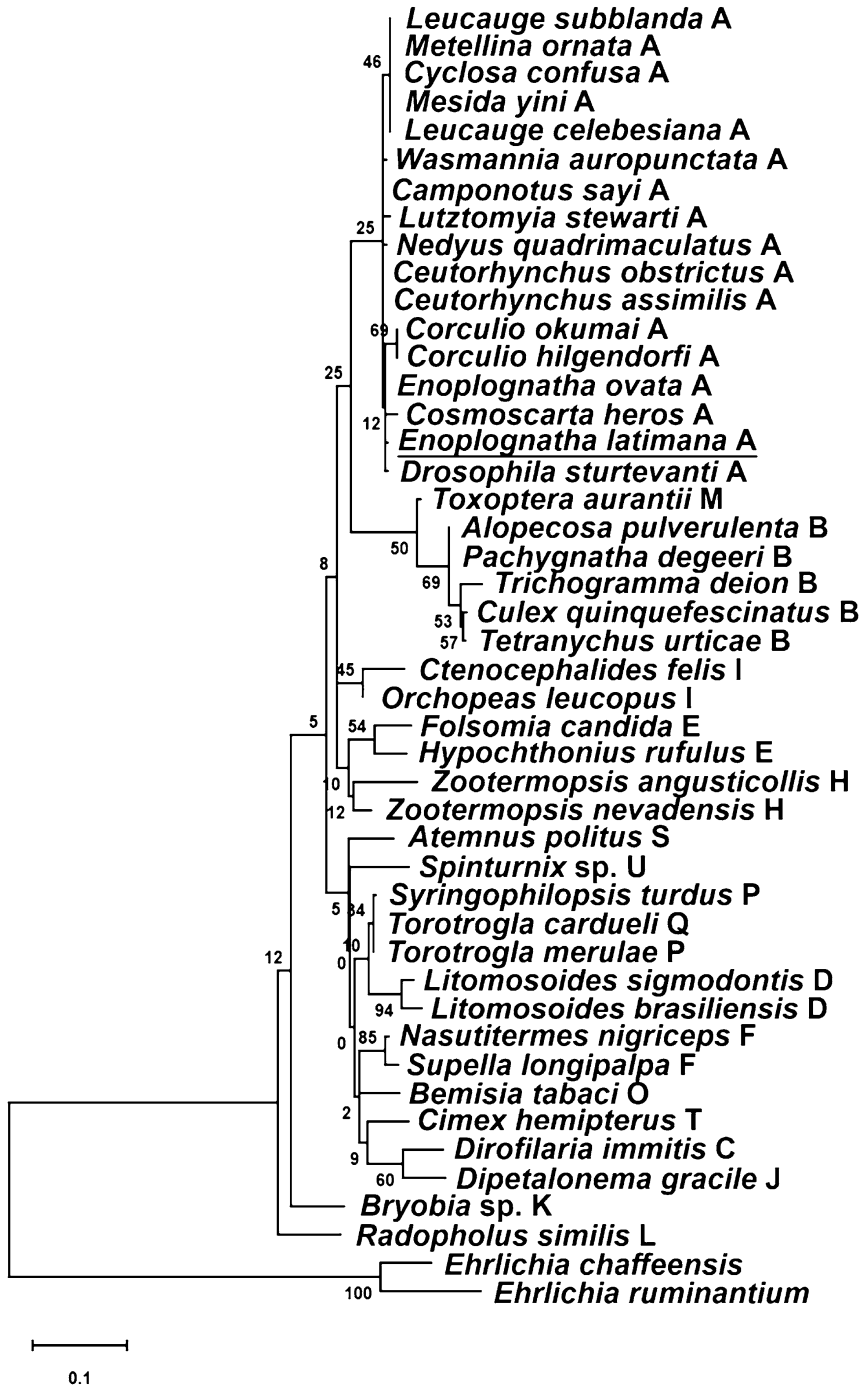
Maximum likelihood reconstruction of *Wolbachia* supergroup phylogeny based on concatenated sequence alignments of eight bacterial loci (16S rRNA, *coxA*, *fbpA*, *ftsZ*, *gatB*, *gltA*, *groEL*, *hcpA*) using MEGA 11 software. Strains are designated by their host names, except for outgroup bacteria. Capital letters indicate individual *Wolbachia* supergroups. Bar, substitutions per nucleotide. Bootstrap values based on 1000 replicates are shown on branches.

We have found a unique sequence (5′-GACTTCG-3′) for *Wolbachia* supergroup A in the *ftsZ* gene. This sequence has been identified in *Wolbachia ftsZ* from various species, including *D. sturtevanti* (accession no. CP050531), *C*. *sayi* (accession no. DQ266387), *Ceutorhynchus assimilis* (Paykull, 1800) (accession no. OU906081), *Ceutorhynchus obstrictus* (Marsham, 1802) (accession no. HM012590), *Cyclosa confusa* Bösenberg and Strand, 1906 (accession no. KX380701), *L*. *celebesiana* (accession no. KX380698), *Leucauge* *subblanda* Bösenberg and Strand, 1906 (accession no. MN202113), *Lutzomyia stewarti* (Mangabeira Fo and Galindo, 1944) (accession no. KJ174694), *M*. *ornata* (accession no. KX380693), *M*. *yini* (accession no. KX380706), *Nedyus* *quadrimaculatus* (Linnaeus, 1758) (accession no. MG987989), and *Wasmannia* *auropunctata* Roger, 1863 (accession no. JX499050). The sequence was not found in the *ftsZ* gene of other *Wolbachia* strains representing supergroups B-U used in this study as comparative material. The location of the above nucleotide sequence was determined at positions 673–679 in reference to *ftsZ* of *Wolbachia* from *D. sturtevanti* (accession no. CP050531). An alignment showing the unique *ftsZ* sequence of *Wolbachia* supergroup A is presented in Fig. 1.

### MLST and phylogenetic analysis using the *wsp* and *ftsZ* genes

**Figure 2:**
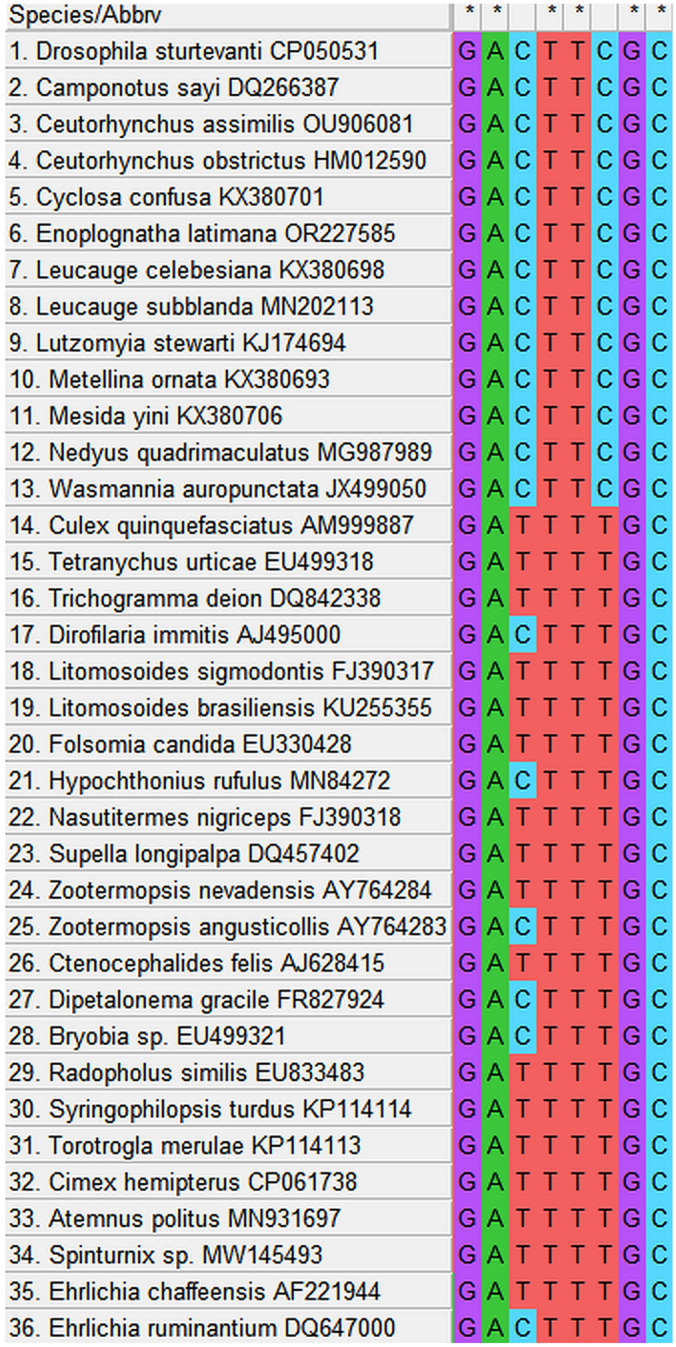
Alignment showing the unique 5′-GACTTCG-3′ sequence from the *ftsZ* gene of *Wolbachia* supergroup A from *Enoplognatha latimana*, *Drosophila sturtevanti*, *Camponotus sayi*, *Ceutorhynchus assimilis*, *Ceutorhynchus obstrictus*, *Cyclosa confusa*, *Leucauge celebesiana*, *Leucauge* *subblanda*, *Lutzomyia stewarti*, *Metellina ornata*, *Mesida yini*, *Nedyus* *quadrimaculatus*, and *Wasmannia* *auropunctata*.

Phylogeny based on concatenated MLST sequence data analysis of eight genes (16S rRNA, *coxA*, *fbpA*, *ftsZ*, *gatB*, *gltA*, *groEL*, and *hcpA*) showed that *Wolbachia* from the spider *E. latimana* was related to endosymbionts of other spider hosts from the order Araneae and dipteran and hemipteran insects, representing supergroup A. The analysis of both individual genes (Supplementary Figs. S1–S8 available in the online Supplementary Information), as well as the combined eight-gene analysis (Fig. 2) consistently demonstrated that the bacterium belonged to supergroup A. The absence of statistically significant evidence of recombination (p = 1.0) using the φ test suggested that *Wolbachia* from *E. latimana* was not a recombinant between strains of other *Wolbachia* supergroups.

**Figure 3:**
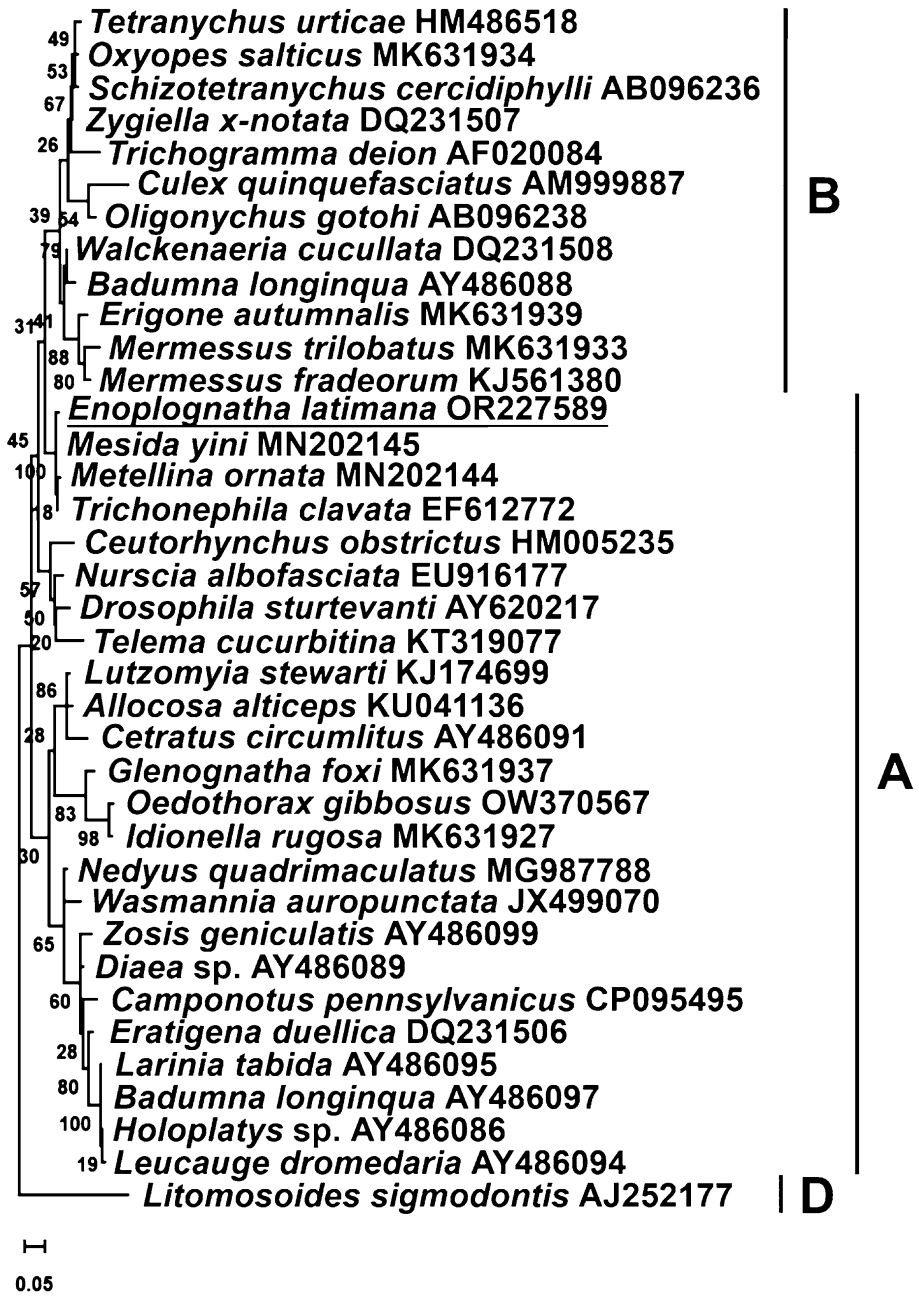
Maximum likelihood reconstruction of *Wolbachia* supergroup phylogeny based on the *wsp* gene sequences using MEGA 11 software. Strains are designated by their host names. *Wolbachia* supergroups (A, B, and D) are indicated. Bar, substitutions per nucleotide. Bootstrap values based on 1000 replicates are shown on branches.

The reconstruction of the phylogenetic tree based on the *wsp* gene of *Wolbachia* supergroups A and B has confirmed that the endosymbiont from *E. latimana* belongs to supergroup A. It formed a cluster with bacteria from three spider species representing the families Tetragnathidae (*M*. *yini* and *M*. *ornata*) and Araneidae (*T*. *clavata*) (Fig. 3).

**Figure 4:**
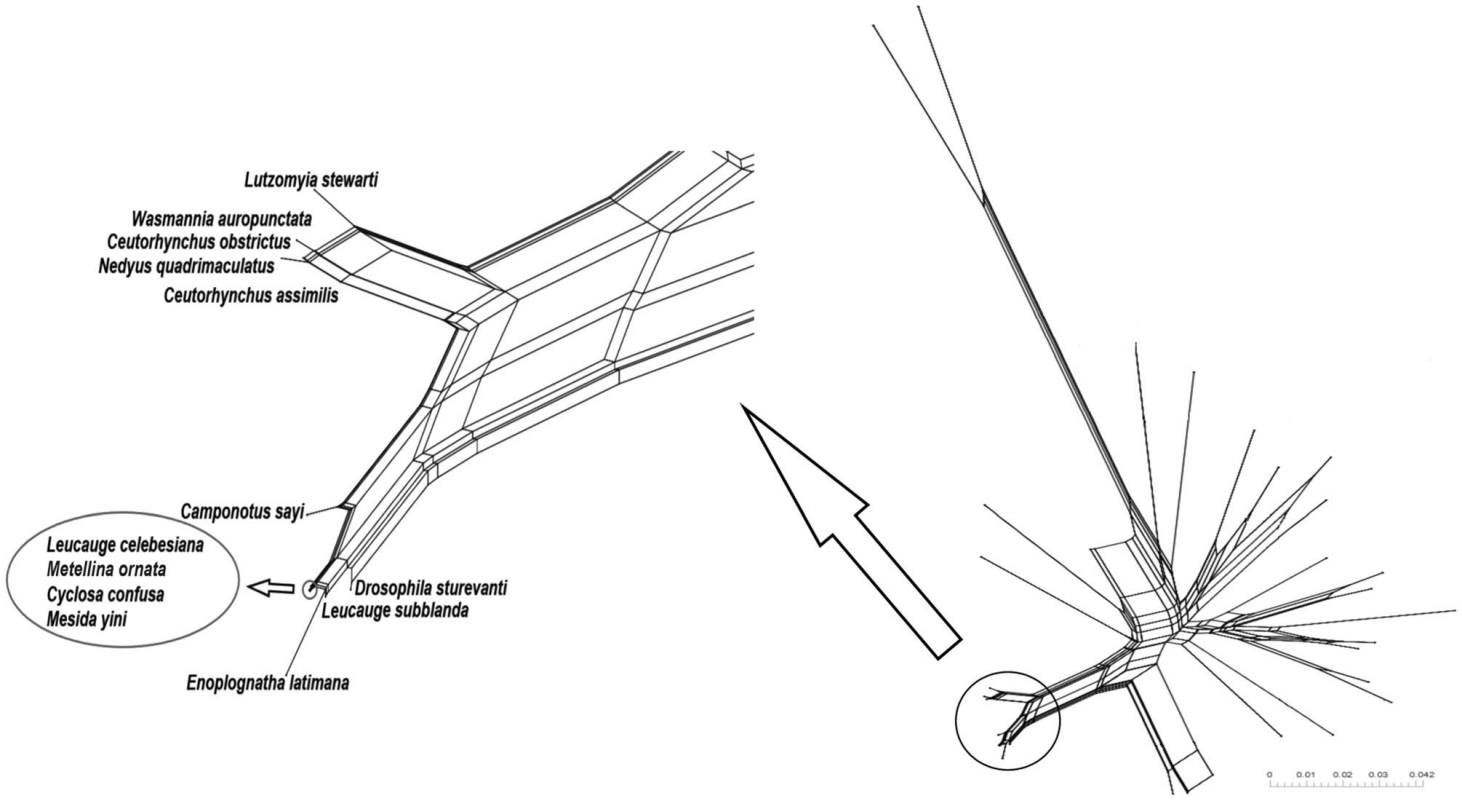
Median network reconstructed for *Wolbachia* supergroup A based on sequence polymorphism of the *ftsZ* genes. Conflicting phylogenetic signals (due to recombination and/or homoplasy) are represented as boxes or parallelograms in the network.

In addition, a phylogenetic network based on the *ftsZ* gene sequences of *Wolbachia* (Fig. 4) revealed the relationship of *Wolbachia* from *E. latimana* with representative strains of *Wolbachia* supergroup A. The network mostly contained very narrow fields, indicating a low level of conflict in the data at the nucleotide level. *Wolbachia* from *E. latimana* clearly clustered with supergroup A strains, excluding other supergroups, as confirmed by the φ test results. Moreover, the analysis indicated the diversity within supergroup A, with two noticeable subgroups: (1) *Wolbachia* from insects *L*. *stewarti*, *W*. *auropunctata*, *C. obstrictus*, *N*. *quadrimaculatus*, *C*. *assimilis*, and (2) *Wolbachia* from spiders *E*. *latimana*, *L*. *celebesiana*, *M*. *ornate*, *C*. *confuse*, *M*. *yini*, *L*. *subblanda*, and insects *C*. *sayi*, *D. sturtevanti*.

Using the PubMLST database, the MLST allelic profile was generated (Table S2). The allelic profile, ST and clonal complex were new according to the available data in the *Wolbachia* database.

## Discussion

The sequences of 16S rRNA, *coxA*, *fbpA*, *ftsZ*, *gatB*, *gltA*, *groEL*, *hcpA*, and *wsp* genes of the bacterial strain found in this study in the spider species *E. latimana* showed the highest identity with *Wolbachia* from supergroup A discovered in other spider hosts and in insects of the orders Diptera and Hymenoptera. The phylogeny based on the concatenated dataset of eight housekeeping genes (16S rRNA, *coxA*, *fbpA*, *ftsZ*, *gatB*, *gltA*, *groEL*, *hcpA*) and the *wsp* gene confirmed the close relationship between *Wolbachia* infecting *E. latimana* and the strains found in other spider and insect hosts, representing the same supergroup carrying the unique 5′-GACTTCG-3′ sequence in the *ftsZ* gene. Additionally, phylogenetic network analysis of the *ftsZ* gene of *Wolbachia* revealed high intragroup diversity of supergroup A, with the supergroup being subdivided into two clades. *Wolbachia* from *E. latimana* clustered with bacteria from other spiders, as well as with strains from dipteran and hymenopteran hosts, further supporting their close relationship. The *Wolbachia* in *E*. *latimana* sequences of *coxA*, *fbpA*, *ftsZ*, *gatB*, and *hcpA* genes did not show an exact match with previously identified STs in the PubMLST *Wolbachia* database and the bacterial strain in the spider is new.

A question arises about the etiology of *Wolbachia* in *E. latimana*, as only one spider out of 39 tested specimen was infected. Spiders of the genus *Enoplognatha* feed on insects of different orders^79–81^, including Diptera and Hymenoptera^82^. Among them, pollinators and other flower-visiting insects are predominant in the spiders diet. Interestingly, our study revealed that *Wolbachia* genes in *E. latimana* exhibited the highest identity and closest relationships to bacteria found in insects, associated with flowering plants, from (1) Diptera: *S. ferrugineus*^83^, *A. albopictus*^84^, and (2) Hymenoptera: *C. sayi*^85^, *C. pennsylvanicus*^86^. Other authors have confirmed that the transfer of *Wolbachia* can occur through the ingestion of remains from infected specimens^87^, and these insects may be a potential source of *Wolbachia* infection in *E. latimana*. It is plausible that *Wolbachia* identified in *E. latimana* could be the result of its presence in insect cells found in the spider’s digestive tract, without infecting the spider’s own cells. In this case, the presence of the bacteria in the spider should be considered accidental rather than as a stable and permanent infection of the host. Considering that hymenopteran and dipteran insects are also parasites of spiders^88^, they could be regarded as potential sources of the bacteria. Some spiders from the genera *Trichonephila*^89,90^ and *Leucauge*^91,92^ are known to be attacked by Hymenoptera parasitoids. Dipteran insects are also known enemies of *Trichonephila* sp. and *Enoplognatha* sp.^93^. *Wolbachia* genes discovered in *E. latimana* showed the highest identity with the corresponding genes of bacterial supergroup A from *T*. *clavata* and *L*. *celebesiana*. Furthermore, the close relationship between these *Wolbachia* strains may suggests the potential possibility of bacterial transmission from insect parasitoids to spider hosts, especially that insect parasites can serve as vectors for *Wolbachia* transmission between hosts^94,95^. While insect parasitoids typically kill their host upon completion of their larval development and parasitism do not allow hosts to transmit the infection to the progeny^88,92,96,97^, there have been cases of spiders that were able to get rid of the intruder and survive^90^. Among the analyzed specimens of *E. latimana*, no insect parasites were found during microscopic observations. If the role of the parasite in the transfer of *Wolbachia* to *E. latimana* may be assumed, one could attempt to speculate that the spider have been temporarily inhabited by the parasite but managed to survive. However, this is not the only potential scenario, as *Wolbachia* transmission via food cannot be ruled out either. Insects from the orders Diptera and Hymenoptera, infected by *Wolbachia* with high genetic identity and relatedness to *Wolbachia* from *E. latimana*, feed on plant nectars. Examples include the flower and leaf nectar-eating dipteran *A*. *albopictus*^84,98^ or the extrafloral nectar-eating hymenopteran *C*. *sayi*^85^. Although literature data do not indicate plant nectar in the diet of *E. latimana* and the possibility of acquisition from nectar contaminated by infected insects is unlikely, it may be not excluded, as some species of spiders, especially early instars of web-building spiders, rely on floral and extrafloral nectar as an important component of their food^99–103^. The latter hypothesis may be supported by the results of other authors, suggesting that food can serve as a medium for *Wolbachia* transmission among invertebrates with similar feeding habits. Sharing the same plant diet may facilitate horizontal transmission of these bacteria^104–106^. All of the modes of *Wolbachia* transfer described above are possible. Although we have not determined the exact etiology of *Wolbachia* in *E. latimana*, the endosymbiont is undoubtedly related to those found in other spider species and insects from the orders Diptera and Hymenoptera, and transfer of the microorganism between these hosts cannot be excluded.

## Conclusion

In conclusion, we have detected for the first time the bacterium *Wolbachia* associated with the spider *E. latimana*. The microorganism was found in only one female and a question arises about the etiology of *Wolbachia* in *E. latimana*. Our data are not sufficient to support the stable presence of *Wolbachia* in the spider species. The high probability of only accidental bacterial presence cannot be excluded. Our study revealed that *Wolbachia* genes associated with *E. latimana* exhibited the highest identity and closest relationships to bacteria found in insects from Diptera and Hymenoptera. As the insects are predominant in the spiders diet, the detected *Wolbachia* could have been present in ingested remains from infected insect specimens.

Our study confirmed the classification of the bacteria associated with *E. latimana* to *Wolbachia* supergroup A. These data provide insight into the occurrence of *Wolbachia* in arthropods. Additionally, we have deposited the sequences of *Wolbachia wsp* and housekeeping genes in publicly available databases, providing valuable molecular data for future comparative studies in this field.

### Supplementary Information

Supplementary Legends.

Supplementary Figure S1.

Supplementary Figure S2.

Supplementary Figure S3.

Supplementary Figure S4.

Supplementary Figure S5.

Supplementary Figure S6.

Supplementary Figure S7.

Supplementary Figure S8.

Supplementary Table S1.

Supplementary Table S2.

## Data Availability

Sequencing data generated and analyzed in this study are deposited to NCBI Nucleotide Database (accession nos. OR220066 and OR227583–OR227590).
