# Supplementary material for: Wolbachia supergroup A in Enoplognatha latimana (Araneae: Theridiidae) in Poland as an example of possible horizontal transfer of bacteria
Source: Sci Rep. 2024 Mar 29;14:7486. doi: 10.1038/s41598-024-57701-y (PMC10980700; doi:10.1038/s41598-024-57701-y)
Supplement: Supplementary file 1 — Supplementary Legends. [file 41598_2024_57701_MOESM1_ESM.docx]

**Supplementary Information**

**Supplementary Table S1.** Primer sets used to characterize *Wolbachia* in *Enoplognatha latimana*.

**Supplementary Figure S1.** Maximum likelihood reconstruction of *Wolbachia* supergroup phylogeny based on the 16S rDNA sequences using MEGA 11 software. Strains are designated by their host names, except for outgroup bacteria. Bar, substitutions per nucleotide. Bootstrap values based on 1000 replicates are shown on branches.

**Supplementary Figure S2.** Maximum likelihood reconstruction of *Wolbachia* supergroup phylogeny based on the *coxA* gene sequences using MEGA 11 software. Strains are designated by their host names, except for outgroup bacteria. Bar, substitutions per nucleotide. Bootstrap values based on 1000 replicates are shown on branches.

**Supplementary Figure S3.** Maximum likelihood reconstruction of *Wolbachia* supergroup phylogeny based on the *fbpA* gene sequences using MEGA 11 software. Strains are designated by their host names. Bar, substitutions per nucleotide. Bootstrap values based on 1000 replicates are shown on branches.

**Supplementary Figure S4.** Maximum likelihood reconstruction of *Wolbachia* supergroup phylogeny based on the *ftsZ* gene sequences using MEGA 11 software. Strains are designated by their host names, except for outgroup bacteria. Bar, substitutions per nucleotide. Bootstrap values based on 1000 replicates are shown on branches.

**Supplementary Figure S5.** Maximum likelihood reconstruction of *Wolbachia* supergroup phylogeny based on the *gatB* gene sequences using MEGA 11 software. Strains are designated by their host names, except for outgroup bacteria. Bar, substitutions per nucleotide. Bootstrap values based on 1000 replicates are shown on branches.

**Supplementary Figure S6.** Maximum likelihood reconstruction of *Wolbachia* supergroup phylogeny based on the *gltA* gene sequences using MEGA 11 software. Strains are designated by their host names, except for outgroup bacteria. Bar, substitutions per nucleotide. Bootstrap values based on 1000 replicates are shown on branches.

**Supplementary Figure S7.** Maximum likelihood reconstruction of *Wolbachia* supergroup phylogeny based on the *groEL* gene sequences using MEGA 11 software. Strains are designated by their host names, except for outgroup bacteria. Bar, substitutions per nucleotide. Bootstrap values based on 1000 replicates are shown on branches.

**Supplementary Figure S8.** Maximum likelihood reconstruction of *Wolbachia* supergroup phylogeny based on the *hcpA* gene sequences using MEGA 11 software. Strains are designated by their host names. Bar, substitutions per nucleotide. Bootstrap values based on 1000 replicates are shown on branches.
