# Supplementary figures and images for: Wolbachia supergroup A in Enoplognatha latimana (Araneae: Theridiidae) in Poland as an example of possible horizontal transfer of bacteria
Source: Sci Rep. 2024 Mar 29;14:7486. doi: 10.1038/s41598-024-57701-y (PMC10980700; doi:10.1038/s41598-024-57701-y)

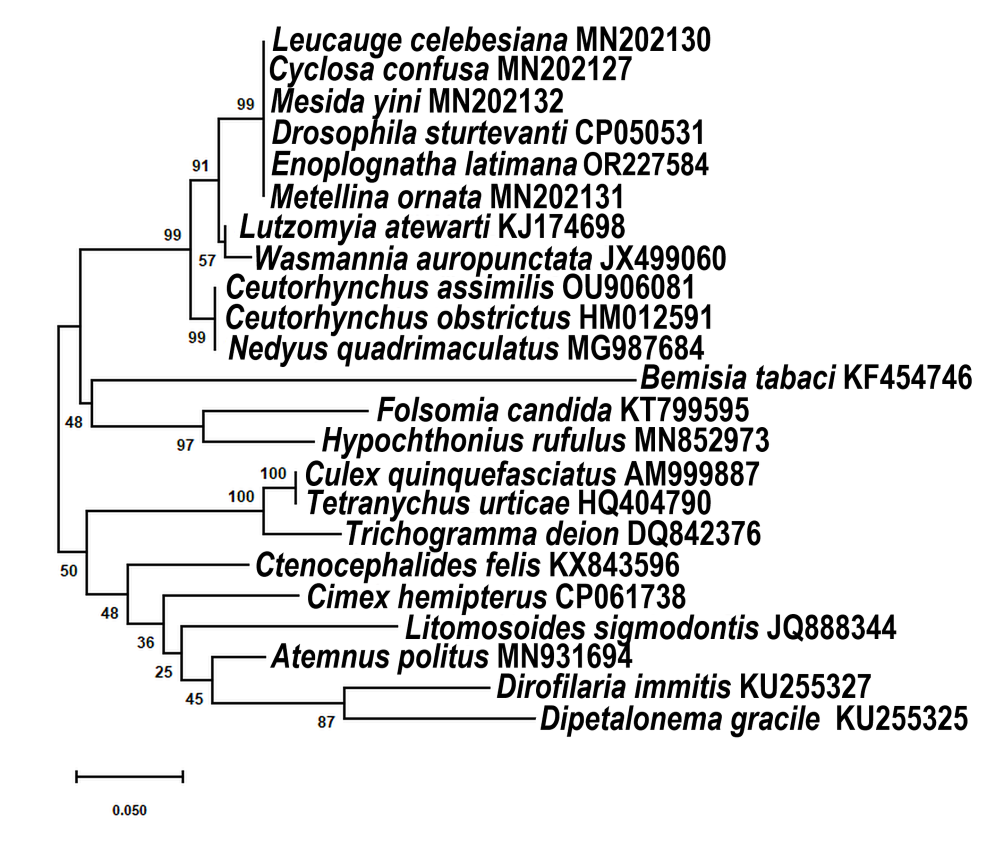

Supplement: Supplementary file 4 — Supplementary Figure S3. [file 41598_2024_57701_MOESM4_ESM.tif]

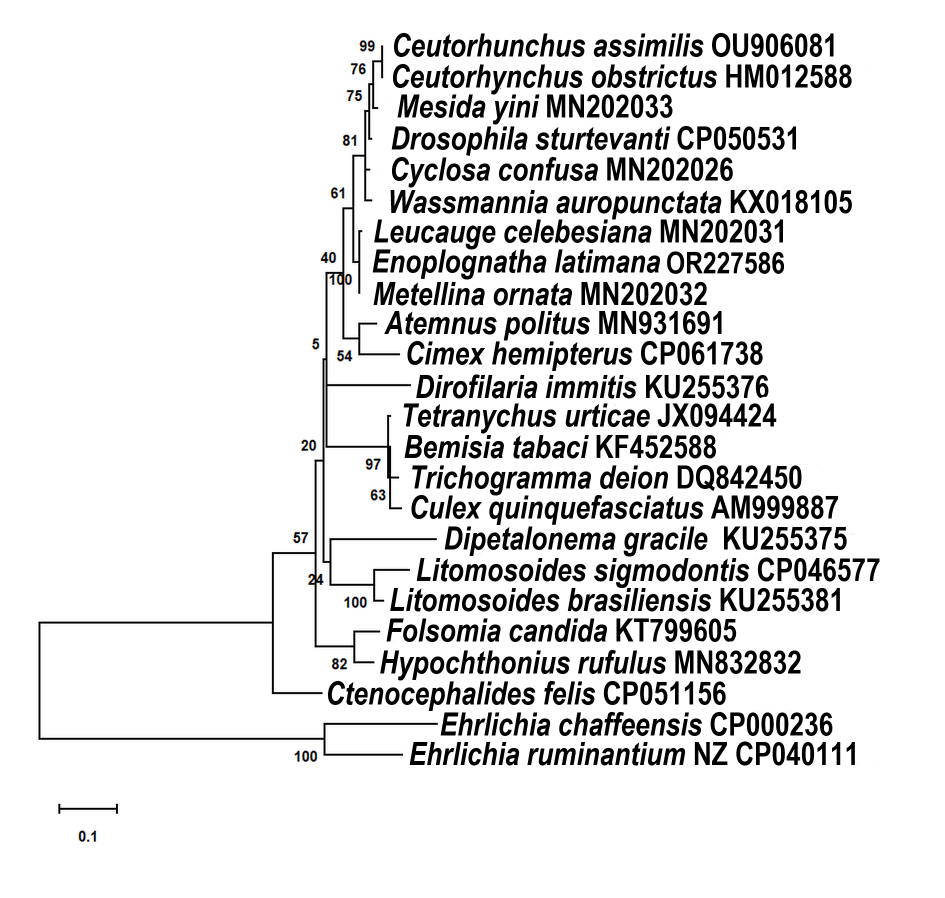

Supplement: Supplementary file 6 — Supplementary Figure S5. [file 41598_2024_57701_MOESM6_ESM.tif]

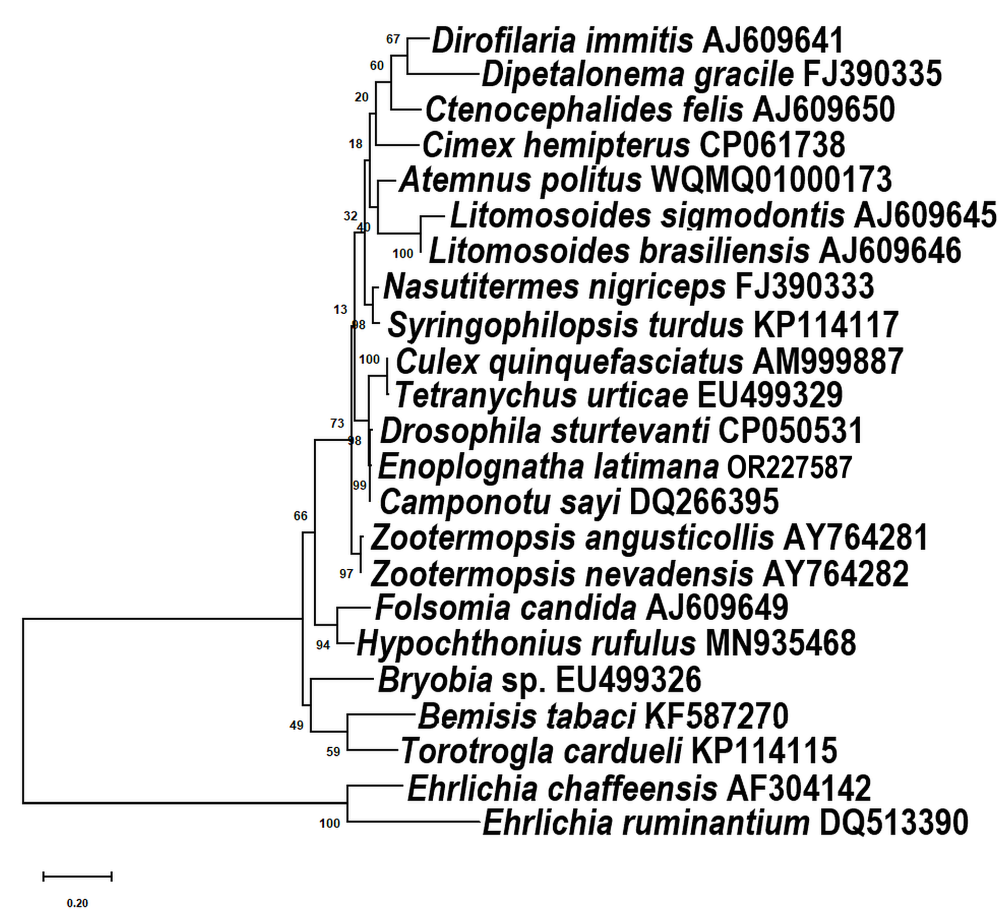

Supplement: Supplementary file 7 — Supplementary Figure S6. [file 41598_2024_57701_MOESM7_ESM.tif]

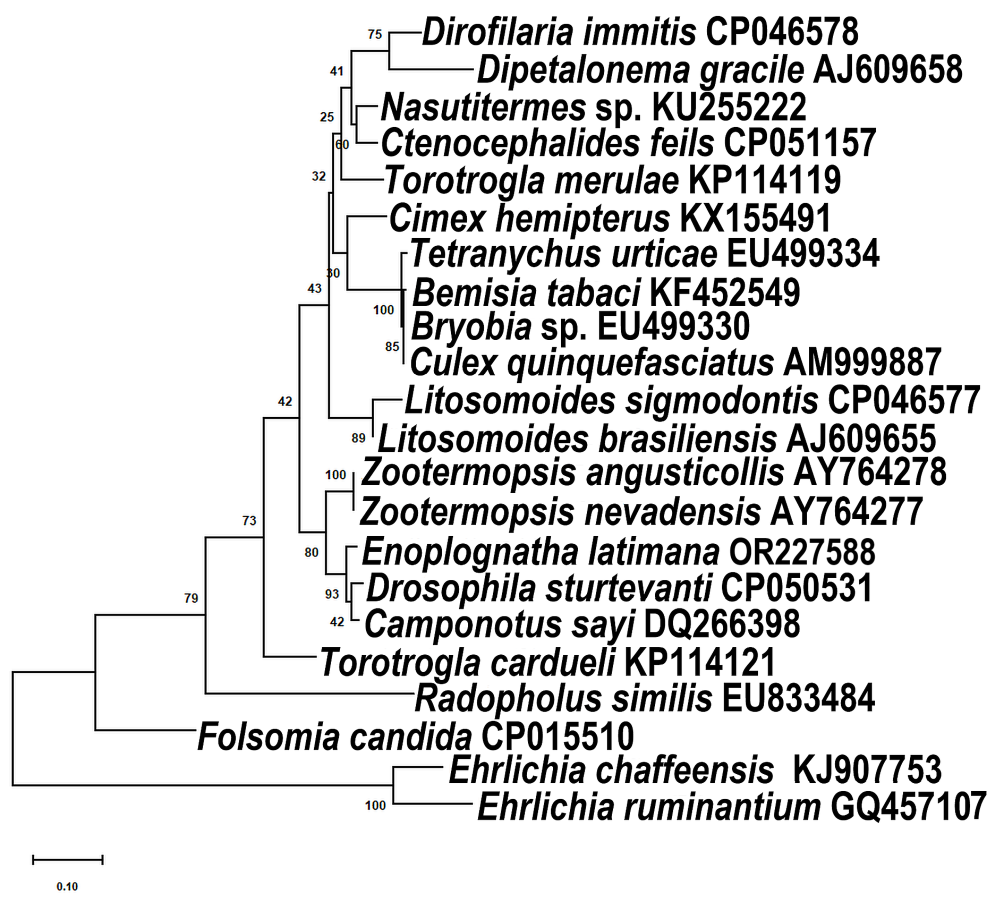

Supplement: Supplementary file 8 — Supplementary Figure S7. [file 41598_2024_57701_MOESM8_ESM.tif]

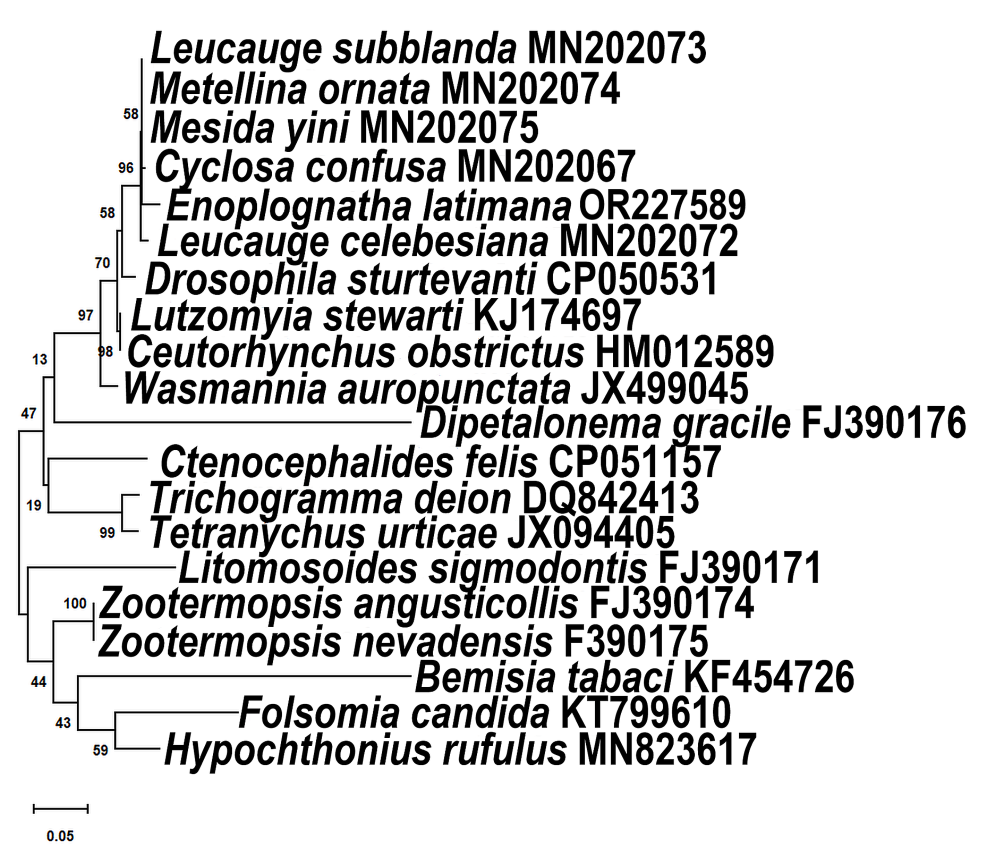

Supplement: Supplementary file 9 — Supplementary Figure S8. [file 41598_2024_57701_MOESM9_ESM.tif]
