# Supplementary material for: Wolbachia supergroup A in Enoplognatha latimana (Araneae: Theridiidae) in Poland as an example of possible horizontal transfer of bacteria
Source: Sci Rep. 2024 Mar 29;14:7486. doi: 10.1038/s41598-024-57701-y (PMC10980700; doi:10.1038/s41598-024-57701-y)
Supplement: Supplementary file 10 — Supplementary Table S1. [file 41598_2024_57701_MOESM10_ESM.docx]

**Supplementary Table S1.** Primer sets used to characterize *Wolbachia* in *Enoplognatha latimana*.

| **Primer name** | **Primer sequence**  **(5’ → 3’)** | **Gene** | **Product** | **Annealing**  **temperature (ºC)** | **Reference** |
| --- | --- | --- | --- | --- | --- |
| EHR16SD  1513R | GGTACCYACAGAAGAAGTCC  ACGGYTACCTTGTTACGACTT | 16S rDNA | 16S rRNA | 55 | [67, 68] |
| EHR16SR  63F | TAGCACTCATCGTTTACAGC  CAGGCCTAACACATGCAAGTC |  |  |  | [67, 69] |
| coxA_F1  coxA_R1 | TTGGRGCRATYAACTTTATAG  CTAAAGACTTTKACRCCAGT | *coxA* | cytochrome c oxidase | 54 | [31] |
| fbpA_F1  fbpA_R1 | GCTGCTCCRCTTGGYWTGAT  CCRCCAGARAAAAYYACTATTC | *fbpA* | fructose-bisphosphate aldolase | 59 | [31] |
| ftsZ_F1  ftsZ_R1 | ATYATGGARCATATAAARGATAG  TCRAGYAATGGATTRGATAT | *ftsZ* | prokaryotic cell division protein | 54 | [31] |
| gatB_F1  gatB_R1 | GAKTTAAAYCGYGCAGGBGTT  TGGYAAYTCRGGYAAAGATGA | *gatB* | glutamyltRNA(Gln) amidotransferase | 54 | [31] |
| hcpA_F1  hcpA_R1 | GAAATARCAGTTGCTGCAAA  GAAAGTYRAGCAAGYTCTG | *hcpA* | conserved hypothetical protein | 54 | [31] |
| wsp_F1  wsp_R1 | GTCCAATARSTGATGARGAAAC  CYGCACCAAYAGYRCTRTAAA | *wsp* | *Wolbachia* surface protein | 59 | [31] |
| WgltAF1  WgltARev1 | TACGATCCAGGGTTTGTTTCTAC  CTCATTAGCTCCACCGTGTG | *gltA* | citrate synthase | 55 | [32] |
| groEL-F  groEL-R | CAACRGTRGSRRYAACTGCDGG  GATADCCRCGRTCAAAYTGC | *groEL* | 60 kDa heat-shock protein | 50 | [70] |
