# Supplementary material for: Wolbachia supergroup A in Enoplognatha latimana (Araneae: Theridiidae) in Poland as an example of possible horizontal transfer of bacteria
Source: Sci Rep. 2024 Mar 29;14:7486. doi: 10.1038/s41598-024-57701-y (PMC10980700; doi:10.1038/s41598-024-57701-y)
Supplement: Supplementary file 11 — Supplementary Table S2. [file 41598_2024_57701_MOESM11_ESM.docx]

**Supplementary Table S2.** MLST allelic profile of *Wolbachia* in *Enoplognatha latimana*.

| **Gene** | **Allelic profile** | **No of nucleotide differences** | **ST** | **Clonal complex** |
| --- | --- | --- | --- | --- |
| *coxA* | 260 | 5 | not defined | not defined |
| *fbpA* | 267 | 0 |  |  |
| *ftsZ* | 231 | 3 |  |  |
| *gatB* | 303 | 0 |  |  |
| *hcpA* | 295 | 6 |  |  |
